# A Large Isolated Hydatid Cyst of the Adrenal Gland: A Case Report and Review of the Literature

**DOI:** 10.1155/2016/9237903

**Published:** 2016-11-28

**Authors:** Fatehi Elnour Elzein, Abdullah Aljaberi, Abdullah AlFiaar, Abdullah Alghamdi

**Affiliations:** ^1^Division of Infectious Diseases, Department of Medicine, Prince Sultan Military Medical City, Riyadh 11159, Saudi Arabia; ^2^Histopathology Department, Prince Sultan Military Medical City, Riyadh 11159, Saudi Arabia; ^3^Urology Department, Prince Sultan Military Medical City, Riyadh 11159, Saudi Arabia

## Abstract

A 44-year-old patient presented with two-year history of (R) lumbar pain. There was a strong history of childhood animals' contact, including dogs. A brother had multiple hydatid cysts requiring surgery. Initial ultrasound showed a large (R) adrenal mass measuring 10 × 9 × 8 cm. Subsequent CT scan confirmed a heavily calcified cyst in the (R) adrenal gland. Hormonal studies were normal. He had an uneventful course following a total adrenalectomy. Isolated adrenal hydatid is extremely rare with an incidence of less than 0.5%; however, the diagnosis should always be suspected in all patients from an endemic area presenting with an adrenal cystic mass.

## 1. Introduction 

Hydatid disease is a zoonotic infection caused by the parasite* Echinococcus granulosus*. Dogs are the principal definitive host while sheep are the most common intermediate one. Individuals get accidentally infected by ingesting the worm's eggs in contaminated food and water, or through close association with domestic dogs. Larvae producing cysts commonly involve the liver, the lungs, and the kidneys. The liver is involved in almost 70% of the cases. Larvae that escape filtration by the liver involve the lungs in 25% of the patients [1]. The disease is distributed throughout the world; it is endemic in the Mediterranean, Eastern Europe, the Middle East, South America, Australia, and South Africa region. Hence, echinococcal infestation should be suspected in any patients from these areas especially in the farming and pastoral locations. Overall adrenal cysts are rare, with a reported autopsy incidence of 0.073% [2], often presenting with broad clinical and radiologic findings, and are thus underrecognized. Occasionally malignant neoplasms greatly mimic benign cysts. As an illustration, only 2 cases (6%) of malignant neoplasms were detected among 31 cystic adrenal lesions diagnosed over a 20-year period (1 epithelioid angiosarcoma and 1 adrenocortical carcinoma) [3]. For this reason, differentiation of cystic adrenal hydatid from other adrenal cysts and adrenal solid tumors with cystic change presents a diagnostic challenge on imaging alone. This is particularly difficult in large sized cysts. One study showed that 1.2% of lesions are malignant, and all exceeded 5 cm [4]. Hydatid cysts account for only 6%-7% of all adrenal cysts. On the other hand, isolated adrenal hydatid cyst constitutes less than 1% of overall hydatid cases [5].

## 2. Case Report

**Figure 1:**
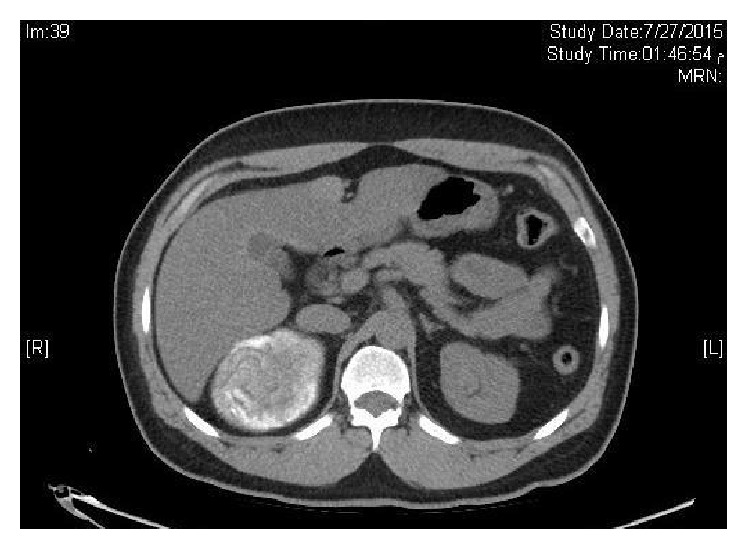
CT scan abdomen showing (R) adrenal mass.

**Figure 2:**
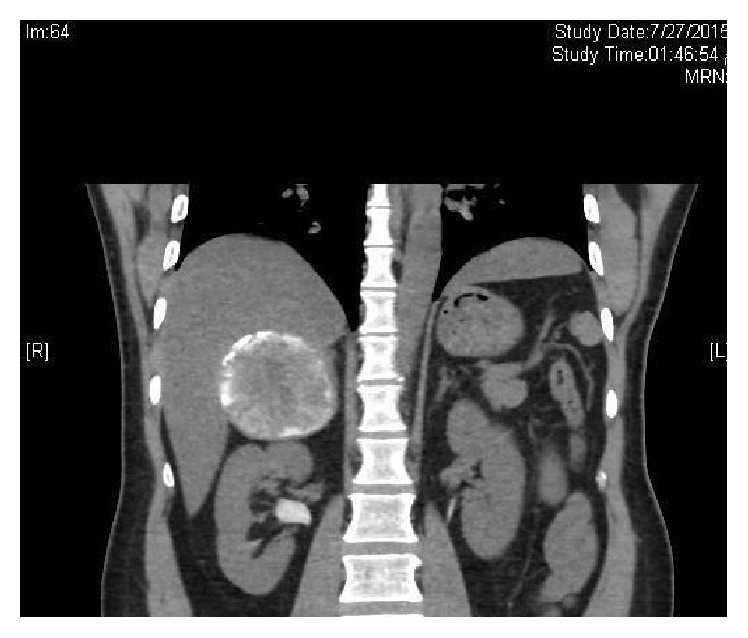
CT scan showing (R) adrenal mass with calcification.

**Figure 3:**
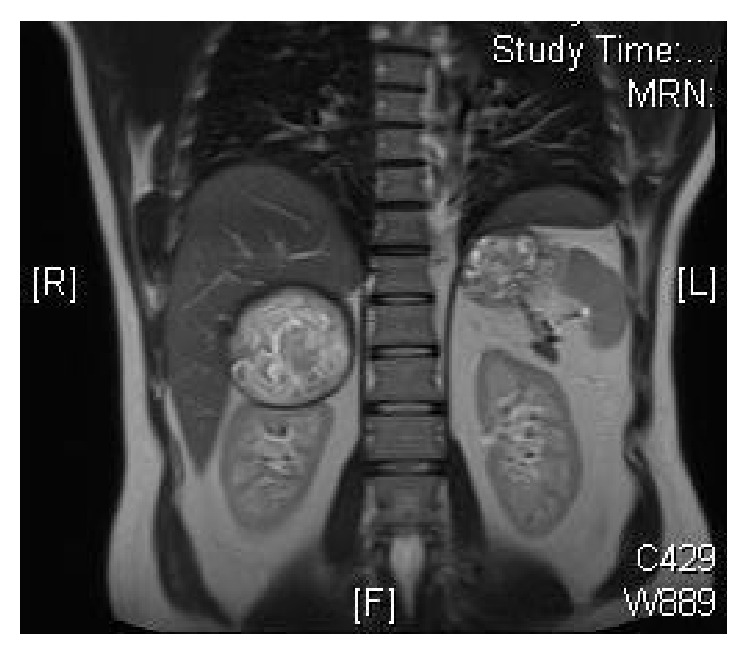
MRI with R adrenal mass.

**Figure 4:**
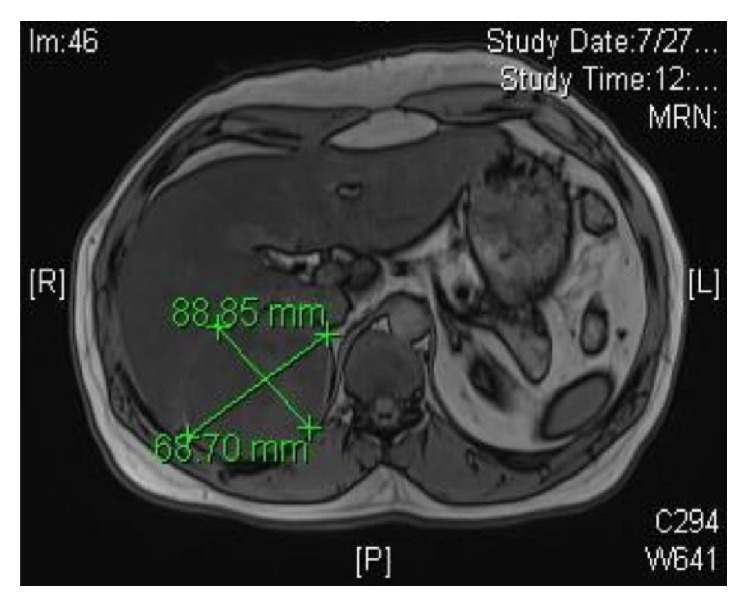
MRI (R) adrenal mass 88 × 68 mm.

**Figure 5:**
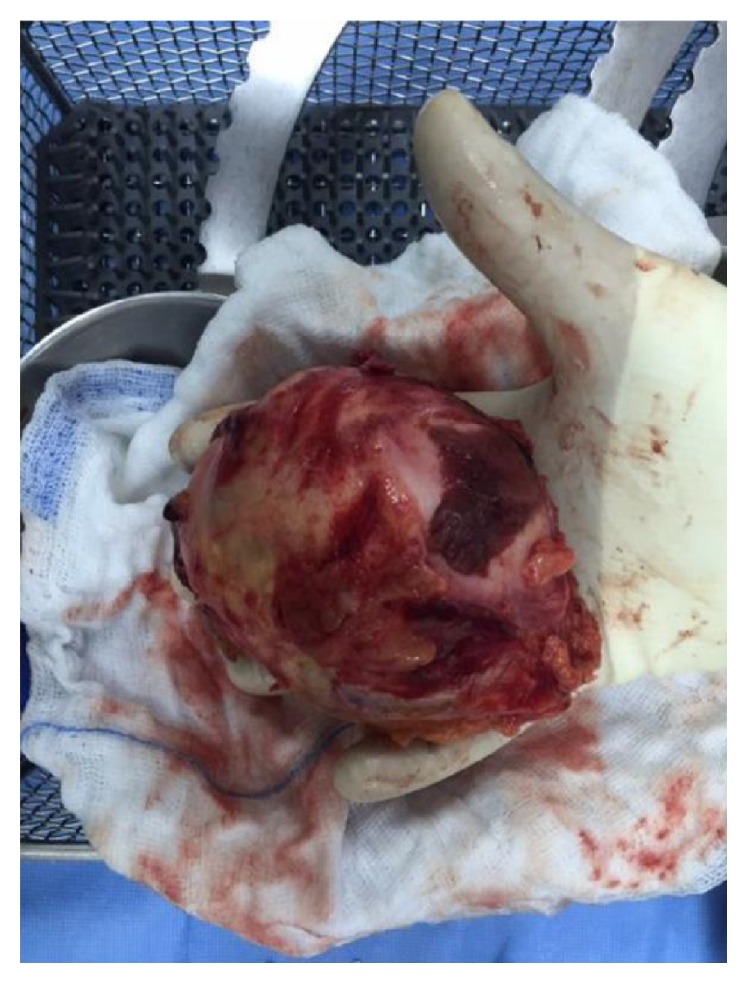
Gross pathology of the resected adrenal gland.

**Figure 6:**
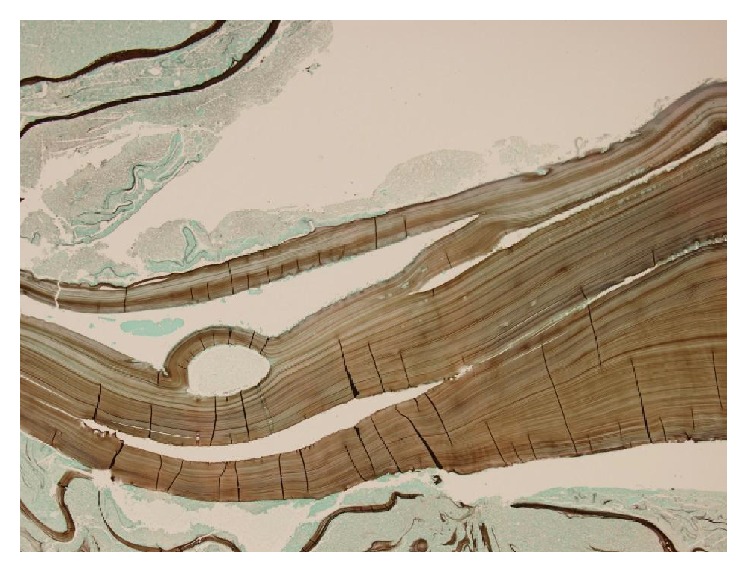
Microscopic image showing characteristic laminated pattern of cyst membrane (GMC stain magnification ×40).

**Figure 7:**
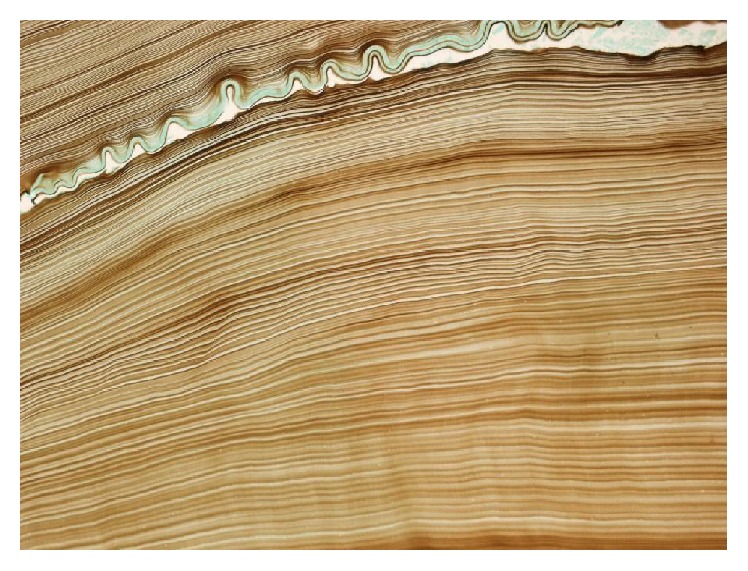
Microscopic image showing characteristic laminated pattern of the cyst membrane (GMC stain magnification ×200).

**Figure 8:**
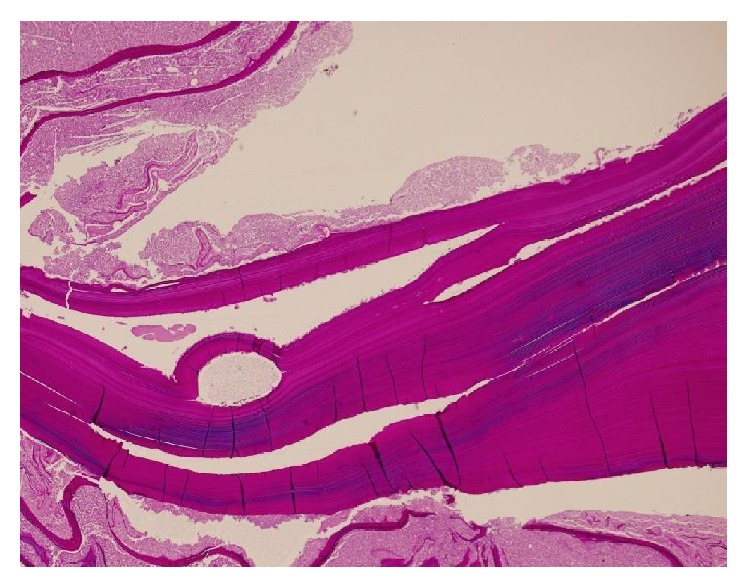
Laminated middle layer of the capsule (PAS stain).

A 44-years-old man was seen in the clinic for recurrent (R) flank pain of 2-year duration. Ultrasound and subsequent CT scan performed 2 years ago showed a (R) suprarenal mass. He denied a history of hypertension, palpitations, or syncopal attacks. Serum cortisol, ACTH, metanephrine, and normetanephrine were normal at 201 nmol/L, 2.8 pmol/L (NR 1.6–13.90), 29 ng/L (NR < 90), and 92.0 ng/L (NR < 129), sequentially. Similarly, aldosterone and renin levels were normal with aldosterone/renin ratio of 3.6. Serum testosterone and dehydroepiandrosterone sulphate were also normal, at 5.94 nmol/L and 3.61 *μ*mol/L successively. He was labeled as a nonfunctioning (R) adrenal mass. He later admitted to a strong history of childhood contact with animals including dogs. His elder brother was operated for multiple hydatid cysts in the abdomen. There were no urinary or other systemic symptoms. General examination including the blood pressure was normal apart from mild tenderness in the (R) renal angle. CT scan abdomen in July 2015 showed a large well-defined oval shaped heavily calcified cystic mass at the right adrenal gland, measuring 8.5 × 6.6 cm on transverse and AP diameter, respectively (Figures 1 and 2). MRI confirmed the CT finding. The mass depicts low T1 and heterogeneous high T2 signal intensity with internal stripes in T2 resembling water lily sign but showed no enhancement. It is surrounded with thin rim of dark T2 signal likely representing calcification (Figures 3 and 4). There was a mass effect on the upper pole of the right kidney and in some areas it appears inseparable from segment VI of the liver. The radiological features were consistent with hydatid cyst. IHA for hydatid was negative at 1 : 80. He was started on albendazole (400 mg twice daily) and praziquantel 600 mg weekly, for four weeks prior to surgery. In view of the persistent loin pain and the large cysts (≥5 cm diameter), he underwent (R) adrenalectomy through a right subcostal incision without spillage of the cyst content. The patient tolerated the procedure well with uneventful postoperative course. Macroscopic appearance showed well circumscribed cystic lesion measuring 10 × 8 × 4.5 cm (Figure 5) with focal multiloculated appearance. Histopathology revealed dense fibrous capsule with three layers showing a middle layer with characteristic lamination pattern and focal calcification. The cyst content is a lightly dense proteinaceous, with a jelly-like matrix material (Figures 6, 7, and 8). No scolices or hooks were seen and no associated granulomas or neoplasia. The histological features were consistent with hydatid cyst. He was finally discharged on albendazole 400 mg BD for another four weeks.

## 3. Discussion 

Hydatid cyst involving the adrenals is rare and is usually a part of a generalized Echinococcosis. It is frequently discovered incidentally. Furthermore and similar to our patient, abdominal pain resulting from organ compression can be a presenting feature [6]. In very rare cases hypertension that subsides with resection of the cyst had mimicked a phaeochromocytoma [7]. The radiological findings in our patient are highly suggestive of hydatid disease despite a negative serology. The presence of solid mass and dense calcification is similar to a type 5 hepatic cyst. WHO classifies hydatid cyst into type 1 with a well-defined, anechoic lesion; type 2 demonstrates the separation of the membrane (the “water lily” sign formed by the undulating membrane); type 3 is characterized by the presence of septa and intraluminal daughter cysts. Type 4 is a nonspecific solid mass while type 5 is characterized by a solid cyst with a calcified wall [8].

Although serology is useful in the diagnosis of hydatid disease, a number of patients may have a negative test. In some series 30–40% of patients with hepatic cystic echinococcosis are antibody negative. This could be due to the capacity of* E. granulosus* antigens to inhibit B cell activity and proliferation [9]. In general, the sensitivity of the serological tests is determined by the location and state of the cysts [10]. The indirect hemagglutination (IHA) test and ELISA have a sensitivity of 80% overall, (90% in hepatic echinococcosis, 40% in pulmonary echinococcosis). Our patient cyst's calcification adds further to the difficulty in the diagnosis. It has been previously reported that false negative serology ensues when the cyst is senescent, dead, or calcified [11]. Consequently, a negative serology does not exclude the diagnosis.

Hydatid cyst can be asymptomatic and need not any intervention except for doubt in the diagnosis and in large cyst causing mass effect. Treatment of adrenal hydatid, when indicated, is mainly surgical and by total cyst excision. Small asymptomatic nonfunctioning cts are treated conservatively. Total adrenalectomy may be considered when the cyst has completely destroyed the gland. Both laparoscopic resection of an adrenal hydatid and laparotomy are accepted surgical intervention [12]. Laparotomy nonetheless allows a better exploration of the peritoneal cavity. Adjuvant albendazole pre- and postoperatively reduced recurrences in hepatic hydatidosis. Of patients who received albendazole therapy for hydatid disease of the liver, no patient had viable cysts at the time of surgery, as compared to 94.45% of the patients who did not receive any preoperative albendazole (*P* < 0.01). The recurrence rate without adjuvant albendazole was 16.66% while no recurrence was seen in patients who received albendazole [13]. It may be argued that our patient calcified hydatid cyst might have indicated dead parasites and hence does not require albendazole therapy. However, peripheral calcification has been described previously in both viable and nonviable cysts [14].

This patient's history of childhood contact with animals and the slowly progressive nature of his adrenal mass together with a history of hydatid in his brother are highly suggestive of hydatid disease. The radiological findings are characteristic though not pathognomonic. Both macroscopic and microscopic features are consistent with adrenal hydatid cyst. A caveat to the diagnosis is the absence of the scolices and/or hooks in the histopathology sections, albeit could be focally present within the specimen but not sampled in the tissue sections or on the glass slides.

Overall, isolated hydatid disease of the adrenals is rare. The diagnosis should be suspected in all patients from or who lived in endemic areas. Surgical excision with either laparotomy or laparoscopic approach remains the intervention of choice in such cases. Adjunctive medical treatment improves the outcome and decreases the recurrence rate.
